# Impact of COVID-19 Outbreak on Stoma Surgery and Stoma Clinic Service: A Retrospective Study at a Single Japanese Referral Hospital

**DOI:** 10.1155/2022/4789775

**Published:** 2022-08-04

**Authors:** Hiroaki Nozawa, Akiko Kawasaki, Chieko Hayashi, Kazushige Kawai, Kazuhito Sasaki, Koji Murono, Shigenobu Emoto, Soichiro Ishihara

**Affiliations:** ^1^Department of Surgical Oncology, The University of Tokyo Hospital, Tokyo, Japan; ^2^Nursing Department, The University of Tokyo Hospital, Tokyo, Japan; ^3^Department of Surgery, Tokyo Metropolitan Cancer and Infectious Disease Center Komagome Hospital, Tokyo, Japan

## Abstract

**Aim:**

The impact of the COVID-19 pandemic on medical practice has been frequently reported from Western countries, but there have been few studies in other areas, especially regarding stoma surgery and stoma care.

**Methods:**

We investigated the numbers of all operations and stoma-related surgeries at our hospital in 2019 and 2020. The cumulative numbers of consultations at our ostomy clinic and patient population stratified by the period of having a stoma were compared between these calendar years. The frequency of ostomy clinic visit by individual patients within the first year after stoma creation and stoma-related complications per consultation were also analyzed.

**Results:**

The number of elective surgeries decreased by approximately 10% from 2019 to 2020, but the numbers of stoma creation and closure procedures did not differ. The total numbers of consultations at our ostomy clinic were also similar between these years. However, the percentage of patients with a stoma for less than a year who visited our ostomy clinic increased from 49.7% in 2019 to 53.5% in 2020, whereas the visitation rate for other patients decreased. Moreover, patients with a stoma for less than a year visited the ostomy clinic more frequently in 2020 (0.42/month) than in 2019 (0.30/month, *p*=0.032). There were fewer grade 2 or more severe peristomal complications in 2020 (11% vs 17% in 2019, *p* < 0.001) at our ostomy clinic.

**Conclusion:**

The COVID-19 outbreak led to a shift in the patient population at ostomy clinics of new stoma patients, which may have resulted in fewer peristomal complications.

## 1. Introduction

SARS-CoV-2 is the virus responsible for COVID-19, causing pneumonia and other symptoms. Its outbreak was first observed in Wuhan, China, in December 2019 and was declared a global pandemic in March 2020 by the World Health Organization. The COVID-19 pandemic has jeopardized healthcare systems worldwide and the capability of providing sufficient quality care to patients. Medical staff faced an urgent need to reorganize treatment paradigms, including surgery, for both COVID-19-positive and -negative patients, i.e., the suspension or postponement of elective noncancer procedures and reduced or completely suspended outpatient clinic services [1, 2]. According to an international study published in June 2020, the overall cancellation rate of elective surgery was projected to be 82% for benign diseases and 38% for cancer during the first peak of the COVID-19 pandemic [3]. Some surgeons themselves developed COVID-19 or had to quit their jobs or take a temporary leave of absence in order to care for their family members [4].

Regarding colorectal surgery, several guidelines from Western countries advocated that an open approach was preferable due to the potential risk of aerosol-transmitted exposure to the SARS-CoV-2 virus generated by pneumoperitoneum in laparoscopic surgery [3, 5]. Moreover, stoma creation was recommended in colorectal surgery aimed at avoiding prolonged hospital stay resulting from anastomotic leakage [5–7]. Indeed, the number of laparoscopic colorectal surgeries transiently decreased, whereas the rate of stoma formation increased in spring and summer of 2020 in the United Kingdom [8]. On the other hand, SARS-CoV-2 can migrate to body fluids in infected patients. Zhang et al. reported that the virus is detectable in the stool of more than 50% of patients diagnosed with COVID-19 regardless of disease severity, and remains longer in the feces than in the nasopharynx and deep throat [9]. The Multidisciplinary Italian Study group for STOmas (MISSTO) recommended that follow-up and elective visits to ostomy care clinics be cancelled and/or postponed [10]. In addition to the behavior of SARS-CoV-2, these precautionary actions can place a higher burden on stoma patients [11].

How surgical treatments were affected by COVID-19 and how to manage medical services in the pandemic have been frequently reported mainly from Western countries, where the mortality rate was high during the first wave in early 2020. On the other hand, Japan and other countries experienced relatively modest outbreaks of COVID-19. However, there is a paucity of studies regarding the impact of COVID-19 prevalence on surgical activities, including colorectal surgery and stoma care, from these countries. Therefore, we investigated stoma-related surgery and stoma management before and after the emergence of COVID-19 in Japan by analyzing data at our institute.

## 2. Methods

### 2.1. Collection of Data on Surgical Operations including Stoma Creation and Closure

The Department of Surgical Oncology at the University of Tokyo Hospital chiefly provides comprehensive surgical treatment for both benign and malignant disease of the small bowel, colon, rectum, and anus, and also performs other general and abdominal surgeries. We counted the numbers of patients who underwent surgical operations at our department in 2019 and 2020, and classified them according to the type of surgery (emergency or elective surgery), type of disease (malignant or benign), and organ. We also counted the numbers of patients who underwent diverting or permanent stoma formation and those who underwent stoma closure with or without resection of bowel segment(s) during the same period. The patients' demographic, clinical, and social backgrounds, such as age, sex, body mass index, serum hemoglobin and albumin levels before surgery, stoma site, disease entity leading to stoma creation, comorbidity, marital status, employment status, and distance from home to ostomy clinic were analyzed.

### 2.2. Practice at an Outpatient Ostomy Clinic

Two certified wound, ostomy, and continence (WOC) nurses have been providing specialized and rehabilitative care for ostomy patients every Wednesday and Friday afternoon at our ostomy clinic using a 12 m^2^ outpatient booth and an adjoining 4 m^2^ toilet facility with handrails. Ostomy patients were assessed by the WOC nurses periodically after discharge. The nurses provide additional opportunities for consultation for patients with a problematic stoma requiring acute care.

Regarding patients who visited our ostomy clinic, monthly and total numbers of patients were counted for the years 2019 and 2020. We also analyzed the frequency of clinic visits per month during the first year for each patient.

Stoma-related complications, such as stoma site infection, stoma necrosis, mucocutaneous separation, stoma obstruction, stoma site bleeding, stoma prolapse, stoma stenosis, parastomal hernia, stomal recession, peristomal fistula, and peristomal dermatitis were scrutinized by reviewing narrative descriptions, comments, and photos available in the medical records. Complications for each visit were graded by the Common Terminology Criteria for Adverse Events classification (CTCAE) version 5 (stoma site infection, stoma necrosis, stoma obstruction, stoma site bleeding, stoma prolapse, and stoma stenosis) [12] or defined by Takahashi et al. based on the CTCAE (mucocutaneous separation, parastomal hernia, stomal recession, peristomal fistula, and peristomal dermatitis) [13].

### 2.3. Statistical Analysis

Differences between continuous and categorical variables were determined using the unpaired *t*-test, Mann–Whitney *U* test, chi-squared test, or Fisher's exact test, where appropriate. A *p* value less than 0.05 was considered to indicate significance. All statistical analyses were performed using the JMP 15.1.0 (SAS Institute Inc., Cary, NC, USA).

### 2.4. Ethics

This study was conducted following approval by the ethics committee of the University of Tokyo Hospital (No. 3252-[13]).

## 3. Results

### 3.1. Numbers of All and Stoma-Related Surgeries

**Figure 1:**
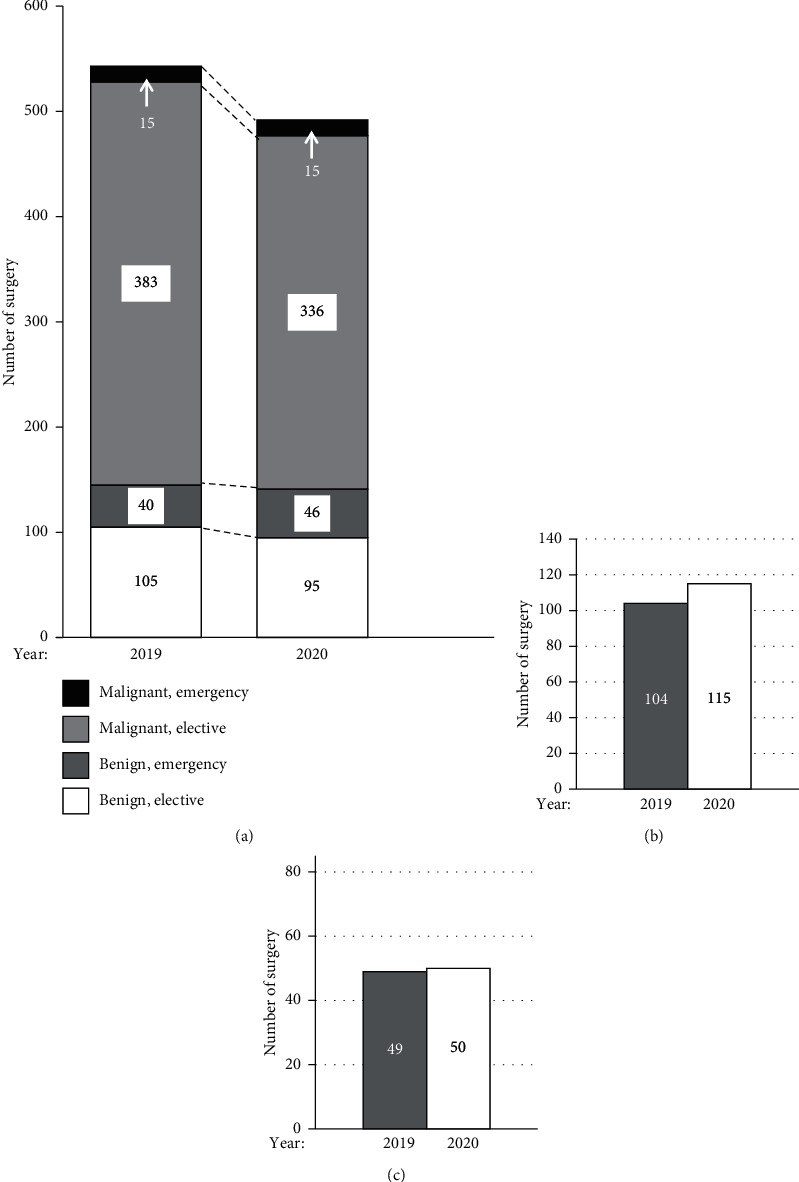
Numbers of all operations and stoma-related surgical procedures according to the calendar year. (a) All operations classified by the type of surgery and disease. The black box indicates emergency operations for malignant diseases, the dark grey box indicates elective operations for malignant diseases, the light grey box indicates emergency operations for benign diseases, and the white box indicates elective operations for benign diseases. (b) Stoma creation. (c) Stoma closure.

At our institute, the total number of patients who underwent surgery was 543 in 2019, which decreased to 492 in 2020. The number of patients who received emergency surgery for benign or malignant disease was similar between these calendar years. On the other hand, the number of elective surgeries for malignancy decreased by 12.3% and that for benign disease by 9.5% in 2020 compared with those in 2019 (Figure 1(a)). As shown in Table 1, the organ distribution of diseases was similar between these calendar years. In addition, there were no differences in other sociodemographic and clinical parameters between the years. A total of 444 bowel operations were performed in 2019 and 411 bowel operations in 2020. The number of patients who underwent stoma creation slightly increased from 104 in 2019 to 115 in 2020 (Figure 1(b)). On the other hand, 49 and 50 patients underwent stoma reversal in 2019 and 2020, respectively (Figure 1(c)). The stoma creation rate in bowel surgery excluding stoma reversal was 26.3% for 2019 and 31.9% for 2020 (*p*=0.094).

The comparative profiles of patients who underwent stoma creation in 2019 and 2020 are shown in Table 2. There was no notable difference in age or sex. Colostomy was newly created in 53 patients and ileostomy in 62 patients in 2020; the distribution of stoma sites did not differ from that in 2019 (*p*=0.72). The majority of diseases that required stoma formation were malignancies of the bowel in both calendar years (78% in 2019 and 76% in 2020); the distribution of other indications for ostomy was similar between 2019 and 2020.

### 3.2. Activity of the Stoma Outpatient Clinic

**Figure 2:**
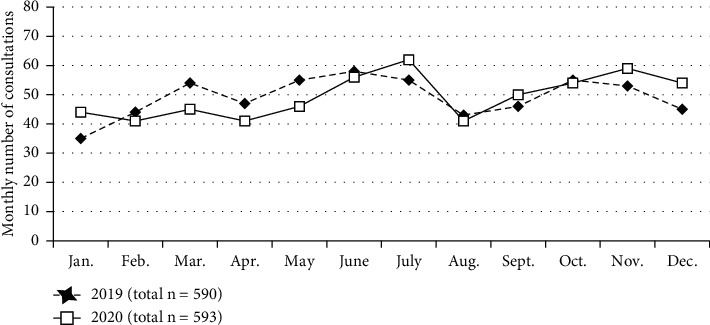
Monthly numbers of consultations at our ostomy clinic in the calendar years 2019 and 2020. Dashed lines indicate the data for 2019, and bold lines indicate the data for 2020.

The cumulative number of patients who visited our outpatient ostomy clinic was 590 in 2019. The number did not change in 2020 (593 patients). The monthly breakdowns are shown in Figure 2. The number ranged from 35 to 62 per month in both calendar years and exhibited similar seasonal variation.

**Figure 3:**
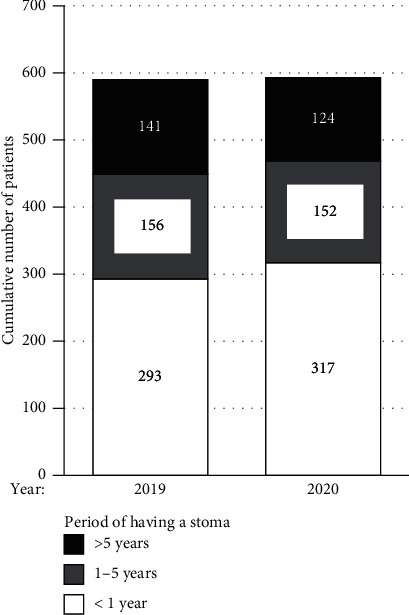
Cumulative number of patients attending our ostomy clinic in the calendar years 2019 and 2020 grouped by the period of having a stoma. White boxes indicate numbers of patients with a stoma for less than one year, striped boxes indicate those with a stoma for 1–5 years, and black boxes indicate those with a stoma for more than five years.

Next, we compared the numbers of patients attending our ostomy clinic grouped by time after stoma creation between 2019 and 2020. Among 590 consultations in 2019, 293 (49.7%) were by patients with a stoma for less than a year, 156 by those with a stoma for 1–5 years (26.4%), and 141 by those with a stoma for more than five years (23.9%), as shown in Figure 3. Compared with these proportions, consultations by patients with a stoma for less than a year slightly increased in 2020 (53.5%), whereas those by the other patient groups decreased (Figure 3).

How often patients having a stoma for less than a year visited our outpatient ostomy clinic was compared between 2019 and 2020. As shown in Table 3, the median frequency in 2020 was 0.42 per month, which was significantly higher than that in 2019 (0.30 per month, *p*=0.032).

Lastly, in order to investigate if the above change in the patient groups affected stoma care outcomes, the rates of stoma-related complications per consultation were compared between 2019 and 2020. The most common complication was peristomal dermatitis in both calendar years; the complication rate markedly decreased from 17% in 2019 to 11% in 2020 (*p* < 0.001, Table 3).

None of patients developed COVID-19 or tested positive for SARS-CoV-2 via transmission at our ostomy clinic (Table 4).

## 4. Discussion

On April 7, 2020, the Government of Japan declared a state of emergency in relation to COVID-19. In our hospital, all elective surgical operations were suspended for one week in mid-April, and step-wisely recovered to the ordinary number as the first outbreak gradually subsided. The decrease in the total number of operations at our hospital reflected these circumstances. In response, we established new standards; all surgical patients were tested for SARS-CoV-2 at admission, and elective surgery for virus-positive patients was cancelled. Temperatures of all hospital visitors were checked by noncontact infrared thermometers, by which febrile outpatients were introduced to a designated fever clinic for the COVID-19 test. The state of emergency was implemented several times thereafter by the government in response to the subsequent domestic COVID-19 waves. However, advanced stage malignancies, imminent and significant perforation causing peritonitis, and inflammatory bowel disease refractory to medical treatments are usually considered categories requiring emergency surgical treatments among colorectal diseases. Thus, elective surgery for SARS-CoV-2-negative patients was postponed on a case-by-case basis, resulting in an approximately 10% reduction in overall elective surgery in 2020 (Figure 1(a)). We also introduced further restrictions such as antigen testing of SARS-CoV-2 for all patients admitted to our hospital.

Similar to the recommendations in Western countries [2, 5, 7], we implemented many infection prevention and control measures in operating rooms. For example, all surgeons and staff other than the anesthesiologists and nurse anesthetists stayed outside of operating theaters during and four minutes after intubation and extubation, as this time is necessary to exchange the air of the theaters completely in our hospital.

Although aerosols produced during pneumoperitoneum in laparoscopic surgery do not originate from the respiratory tract, SARS-CoV-2 can be present in peritoneal fluids [14]. However, the transmission risk via this route is considered very low [15] and the Japan Surgical Society mentioned that there is no clear evidence to support open surgery over minimally invasive surgery [1]. Considering the widely accepted benefits of laparoscopy, we did not replace laparoscopy by laparotomy for presumably SARS-CoV-2-negative patients as long as the aforementioned measures were adopted.

We did not alter our criteria for stoma creation after the COVID-19 outbreak, which is why the number and rate of stoma creation did not significantly differ between 2019 and 2020 at our hospital (Figure 1(b)). There are at least two underlying reasons: a relatively low rate of anastomotic leakage in laparoscopic anterior resection in Japan (8.6% by the recent nationwide database [16] and 2.3% at our hospital [17]) and that the Japan Surgical Society has no recommendations for stoma formation in colorectal surgery.

Stoma-related complication rates depend on ostomy type [18]. The frequency of peristomal dermatitis, which is more frequently observed in ileostomy than colostomy [19], dramatically decreased from the year 2019 to 2020, although the proportion of new patients with ileostomy did not change during the study period at our hospital. In addition, we did not find any changes in other clincopathological and social parameters between these calendar years, which may not only have influence ostomy clinic visits and/or postoperative complications but also have been affected by the COVID-19 outbreaks (Table 1). Moreover, two specialized WOC nurses were not redeployed to other roles in response to the emergence of COVID-19, and continued to provide stoma care throughout the study period. Therefore, we consider that the change in the complication rate may have been attributable to the COVID-19 outbreaks.

After the emergence of COVID-19, the Japanese Society of Stoma and Continence Rehabilitation advocated the guidelines regarding consultation at ostomy clinics; consistent with the recommendation in other countries [10], they include rethinking of the indications for ostomy clinic visits and seeking alternative methods and risk assessment based on PCR testing of SARS-CoV-2, stoma site, stool form scale, patient posture, zoning of SARS-CoV-2-positive and -negative patients, and additional use of personal protective equipment such as single-use gowns, surgical masks, hair-nets, eye protection, and disposable gloves. Stoma care service at our clinic abides by the abovementioned guidelines. Two WOC nurses have been running our ostomy clinic with a limited capacity in terms of space and time. They telephoned ostomy patients and discussed the necessity of attending clinics prior to their scheduled visits. As a result, the frequency of clinic visits by patients having a stoma for more than a year decreased after the COVID-19 outbreak, whereas the released reservation frames were fully utilized for the care of new ostomy patients. Ostomy patients with experience and confidence in care may have refrained from attending our outpatient clinic during the COVID-19 outbreak. The greatest risk of ostomy-related complications is in early postoperative years [20]. Persson et al. described that the majority of complications, with peristomal dermatitis being the most frequent, are usually observed in the first 3 months in ileostomy and in the first 6–12 months in colostomy [18]. Therefore, the changes in ostomy clinic visit observed in 2020 were considered rather favorable for new ostomy patients in need of intensive stoma care. Importantly, there was no documented case of ostomy clinic-mediated spread of COVID-19, suggesting that our proactive precautions were effective for avoiding the transmission of SARS-CoV-2 among patients and medical staff through stoma care.

Our findings from a single institute should be interpreted with caution, as our situation may not represent the totality of hospitals in Japan. In addition, this retrospective study has several limitations. Early postoperative care in the ward before discharge was not included. There may be several patients too ill to visit our ostomy clinic. As stoma conditions at home were not assessed and follow-up intervals depended on individuals, the complication rates reported here may contain biases. Lastly, we did not evaluate the quality of life of stoma patients in relation to stoma-related complications such as by using questionnaires.

In conclusion, the number of elective colorectal surgery decreased by approximately 10%, whereas those of stoma-related surgeries and the characteristics of new ostomy patients remained unchanged after the emergence of SARS-CoV-2 in a Japanese referral hospital. In response to the COVID-19 outbreaks, the patient profile of an outpatient ostomy clinic with limited resources and capacity was altered accompanied by a reduced rate of peristomal dermatitis. Namely, the overall number of stoma clinic visits was unchanged, but more frequent care was delivered to novices regarding stoma care. Crises sometimes drive innovative methods in healthcare [21]. In addition to the current clinic activity, online remote consultation for stoma care may function as triage for face-to-face outpatient clinics, and provide advantages in terms of saving time and reducing healthcare costs. We hope that our experience will be informative for medical staff in similar situations worldwide.

## Figures and Tables

**Table 1:** Number of operations according to organ.

Calendar year	2019	2020	*p* value
Total number	543 (100%)	492 (100%)	
Organ			
Rectum	151 (27.8%)	111 (22.6%)	0.41
Colon	218 (40.1%)	211 (42.9%)	
Ileum	58 (10.7%)	71 (14.4%)	
Jejunum	5 (0.9%)	4 (0.8%)	
Rectum + colon	10 (1.8%)	11 (2.2%)	
Colon + ileum	2 (0.4%)	62 (12.6%)	
Others	99 (18.2%)	81 (16.5%)	

**Table 2:** Profile of patients who underwent stoma creation.

Calendar year		2019	2020	*p* value
Number of new stoma patients		*n* = 104	*n* = 115	
Variable				
Age, year	Mean ± SD	60.3 ± 14.1	62.5 ± 14.9	0.26

Sex	Male	67 (64%)	68 (59%)	0.42
	Female	37 (36%)	47 (41%)	

Body mass index, kg/m^2^	Mean ± SD	22.8 ± 4.1	22.2 ± 3.7	0.29
Hemoglobin, g/dl	Mean ± SD	12.0 ± 2.4	11.6 ± 2.1	0.16
Albumin, g/dl	Mean ± SD	3.5 ± 0.7	3.4 ± 0.7	0.19

Stoma site	Colostomy	54 (52%)	53 (46%)	0.72
	Ileostomy	49 (47%)	62 (54%)	
	Jejunostomy	1 (1%)	0 (0%)	

Disease for stoma creation	Malignant tumor of the rectum	69 (66%)	66 (57%)	0.85
	Malignant tumor of the colon	11 (11%)	20 (17%)	
	Malignant tumor of the small bowel	1 (1%)	2 (2%)	
	FAP with cancer	4 (4%)	4 (4%)	
	Colitis-associated cancer	4 (4%)	5 (5%)	
	Refractory UC	8 (8%)	4 (4%)	
	Bowel perforation	5 (5%)	7 (7%)	
	Bowel obstruction	1 (1%)	4 (4%)	
	Fistula in the rectum	0 (0%)	2 (2%)	
	Bleeding from diverticula	1 (1%)	1 (1%)	

Comorbid illness	Ischemic heart disease	2 (2%)	8 (7%)	0.11
	COPD	1 (1%)	2 (2%)	1.00
	Chronic hepatitis/cirrhosis	7 (7%)	7 (6%)	1.00
	CKD	1 (1%)	5 (4%)	0.22
	Diabetes mellitus	11 (11%)	20 (17%)	0.15
	Cerebrovascular disease	3 (3%)	11 (10%)	0.054
	Dementia	1 (1%)	3 (3%)	0.62
	Autoimmune disease	6 (6%)	3 (3%)	0.31

Marital status	Single	32 (31%)	28 (24%)	0.83
	Married	58 (55%)	66 (57%)	
	De facto marriage	3 (3%)	2 (2%)	
	Divorced	4 (4%)	3 (3%)	
	Widowed	5 (5%)	11 (10%)	
	Unknown	2 (2%)	5 (4%)	

Employment	Employed	59 (57%)	43 (37%)	0.093
	Taking sick leave	2 (2%)	7 (6%)	
	Receiving social security	12 (11%)	10 (9%)	
	Pensioner	17 (16%)	30 (26%)	
	Proprietor	7 (7%)	8 (7%)	
	Student	1 (1%)	1 (1%)	
	Housewife	6 (6%)	16 (14%)	

Distance from home to clinic, km	Mean ± SD	13.3 ± 12.0	13.3 ± 11.8	0.98

SD, standard deviation; FAP, familial adenomatous polyposis; UC, ulcerative colitis; COPD, chronic obstructive pulmonary disease; CKD, chronic kidney disease; CNS, central nervous system.

**Table 3:** Frequencies of ostomy clinic visit during the initial 12 months after stoma formation.^†^

Calendar year	2019	2020	*p* value
Number of new stoma patients	*n* = 104	*n* = 115	
Frequencies of ostomy clinic visit (per month)	0.30 (0–1.25)	0.42 (0–1.67)	0.032

^†^Counted for the period with an ostomy.

**Table 4:** Stoma-related complications per consultation at outpatient ostomy clinic according to the year.

Calendar year	2019	2020	*p* value
Total cumulative number of consultations	*n* = 590	*n* = 593	
Complication^†^			
Stoma site infection	0 (0%)	0 (0%)	1.00
Stoma necrosis	0 (0%)	0 (0%)	1.00
Mucocutaneous separation	1 (0%)	4 (1%)	0.37
Stoma obstruction	0 (0%)	0 (0%)	1.00
Stoma site bleeding	1 (0%)	1 (0%)	1.00
Stoma prolapse	6 (1%)	9 (1%)	0.61
Stoma stenosis	0 (0%)	1 (0%)	1.00
Parastomal hernia	5 (1%)	8 (1%)	0.58
Stomal recession	4 (1%)	1 (0%)	0.22
Peristomal fistula	3 (1%)	1 (0%)	0.37
Peristomal dermatitis	103 (17%)	49 (11%)	<0.001

^†^Grade 2 or severer complications were counted.

## Data Availability

The datasets generated and/or analyzed during the current study are not publicly available because they are derived from the patient database of the hospital and hence subject to confidentiality but are available from the corresponding author on reasonable request.
